# Social capital and frequent attenders in general practice: a register-based cohort study

**DOI:** 10.1186/s12889-018-5230-2

**Published:** 2018-03-02

**Authors:** Alexander A. Pasgaard, Maiken H. Mæhlisen, Charlotte Overgaard, Linda Ejlskov, Christian Torp-Pedersen, Henrik Bøggild

**Affiliations:** 1 Public Health and Epidemiology Group, Department of Health Science and Technology, Aalborg University, Niels Jernes Vej 14, DK-9220 Aalborg East, Denmark; 2 Unit of Epidemiology and Biostatistics, Aalborg University Hospital, Sdr. Skovvej 15, DK-9000 Aalborg, Denmark

**Keywords:** Primary health care, General practitioners, Health care utilization, Social capital

## Abstract

**Background:**

Frequent attendance to primary care constitutes a large use of resources for the health care system. The association between frequent attendance and illness-related factors has been examined in several studies, but little is known about the association between frequent attendance and individual social capital. The aim of this study is to explore this association.

**Methods:**

The analysis is conducted on responders to the North Denmark Region Health Profile 2010 (*n* = 23,384), individually linked with information from administrative registers. Social capital is operationalized at the individual level, and includes cognitive (interpersonal trust and norms of reciprocity) as well as structural (social network and civic engagement) dimensions. Frequent attendance is defined as the upper-quartile of the total number of measured consultations with a general practitioner over a period of 148 weeks.

**Results:**

Using multiple logistic regression, we found that frequent attendance was associated with a lower score in interpersonal trust [OR 0.86 (0.79–0.94)] and social network [OR 0.88 (0.79–0.98)] for women, when adjusted for age, education, income and SF12 health scores. Norms of reciprocity and civic engagement were not significantly associated with frequent attendance for women [OR 1.05 (0.99–1.11) and OR 1.01 (0.92–1.11) respectively]. None of the associations were statistically significant for men.

**Conclusion:**

This study suggests that for women, some aspects of social capital are associated with frequent attendance in general practice, and the statistically significant dimensions belonged to both cognitive and structural aspects of social capital. This association was not seen for men. This indicates a multifaceted and heterogeneous relationship between social capital and frequent attendance among genders.

**Electronic supplementary material:**

The online version of this article (10.1186/s12889-018-5230-2) contains supplementary material, which is available to authorized users.

## Background

### Social capital and frequent attenders in general practice

Social capital has been associated with a diverse range of health outcomes over the last 20 years, with many studies focusing on all-cause mortality [1–3]. However, the causal mechanism for this association is complex and poorly understood [4, 5], which emphasizes the need for more research into the phenomenon. One of the suggested pathways for social capital to interact with health is through health care access and utilization [6–8]. Social capital is a complex theoretical construct with a very convoluted genealogy [9], here we understand social capital as those actual or potential resources, such as advice, care, financial and emotional support and more, that are available to the individual through his or her social network. In the context of health care utilization, we thus expect more close-knit ties, to exert a larger influence on behavior as we suspect that health is often discussed with family and other close confidents, leading to use of informal resources, which potentially reduces the need for formal health care use.

However, the relationship between social behavior and health care utilization is complex [8]. A review of the association between health care utilization and social capital found little consistency in the findings of the investigated studies, which suggest that this might be due to the lack of a theoretical framework in the approach to working with social capital. The findings propose that future studies should seek to adhere to a strong theoretical framework, and suggest that more studies using longitudinal data are needed to explore the connection [6]. One possible way of measuring utilization is focusing on the users with frequent attendance in general practice. Frequent attendance is defined as a disproportionate amount of general practice consultations compared to the general populace [10]. Frequent attendance has naturally been associated with illness [10] but also with psychological and social factors [11], but the association with social capital has not been explicitly examined. Attendance not associated to health constitutes a potential misuse of general practice resources, and we hypothesize that social capital might provide fundamental supportive resources that mitigate both health and non-health-related use of the general practice, thus lowering the risk of becoming a frequent attender.

### Conceptual operationalization of social capital

Contemporary social capital literature is predominately inspired by the works of Robert Putnam and Pierre Bourdieu [6, 9, 12–15]. Bourdieu defined social capital as “…the aggregate of the actual or potential resources which are linked to possession of a durable network of more or less institutionalized relationships of mutual acquaintance and recognition…” [12], while Putnam’s contribution focused on the civic and area level aspects of social capital [13, 15]. Putnam’s operationalization is used extensively in constructing a quantifiable measure for social capital, while the level of analysis and interpretation is largely inspired by Bourdieu. Social capital is often disaggregated into subdimensions characterized by the aspects of social life they seem to relate to. The conceptualization of these dimensions may be based on whether they are measured as structural or cognitive items, and the dimensions serve the purpose of separating “what people do” from “what people think” [16]. In the current study, the structural dimension is further separated into formal and informal social relations, while the cognitive dimension is separated into trust and perceived norms of reciprocity. The subcategorization serves multiple purposes, one of which is providing results that maintain heuristic value for future public health policies. The subcategories will allow for a more targeted approach to policies, provided they differ in the strength and direction of effect. Studies have indicated that social capital is a multidimensional concept and that the subdimensions might not correlate in all circumstances [17]. Another benefit is that the sub dimensions allow for interstudy comparability, even in case of differences in the theoretical foundation of the studies. This study includes dimensions that explore both the size and frequency of association of the individual’s network, but also the perceived benefits inherent in these. Other authors might operationalize this dichotomy differently, and keeping the dimensions separate allows for results that are independent of this theoretical choice. The dimensions explored in the current study were suggested by Ejlskov et al. and correspond to those used in previous literature [14, 16, 18].

### Measuring frequent attendance in a Danish context

The Danish health care setting offers a unique opportunity to examine health care utilization in different groups. Due to the Danish health care system being publicly financed [19], the financial barrier to general practice consultations is mostly nonexistent, and thus any difference in the proportion of frequent attenders must be explained by other factors than economy. Commonly, the first point of contact with the health care system will be at the general practice level, with the exception of emergency and dental care. For this reason general practice consultations are a useful measure for health care services used. The exhaustiveness of the Danish national administrative registers allows accounting for a number of potential confounders, which may lead to new insight into the suspected association between social capital and frequent general practice attendance.

Previous literature has shown that men and women have different patterns of general practice use [10, 20]. Another study has shown that social capital’s association with health behavior differs between genders [21]. Acknowledging this evidence, we hypothesize that the association between social capital and health care utilization might differ by gender. Recent literature indicates that multiple complex mechanisms might be responsible for the observed differences in health seeking behavior between genders, which might in part be explained by certain traditional notions of masculinity, that places negative value on help-seeking [22, 23]. However in a systematic literature review related to health care utilization by men, emphasis was also placed on emotions pertaining to help-seeking such as fear, embarrassment and vulnerability/lack of control, as well as a tendency to view experienced symptoms as insignificant and with a poor relationship to the health care professional [23].

Social capital is closely related to other psychosocial pathways to health. A recent systematic review found that social capital might both be correlated to and a mediator/buffer for the perceived effects of socioeconomic status on health [24]. Uphoff et al. find that 56 of the 60 studies identified in the review, show a correlation between socioeconomic status, social capital and health [24]. To disentangle the main effects of social capital from those of socioeconomic status, it is then necessary to consider potential confounders in the analysis. Harpham et al. suggest that income, education and gender should be considered for the analysis [16]. We furthermore include age as a potential confounder, as age is naturally linked to health and has been identified as a determinant of health behavior [23]. While the exact operationalization might differ; income, education, gender, age and self-reported health have commonly been included in previous literature [25].

By definition, health and health care are linked entities. Thus, to investigate the relationship between social capital and frequent attendance in general practice, we need to devise a method of adjusting for the current health of the individual. As any formal diagnosis is necessarily preceded by the use of health care services, we chose to use self-reported health as our health proxy to prevent frequent attendance from biasing towards poor health.

## Methods

### Aims and hypotheses

Based on the literature, we have tested two hypotheses in the analysis:

1. Low individual-level social capital is associated with a high number of consultations in general practice.
2. Different aspects of social capital are heterogeneously associated with frequent attendance.

Further, we aim to investigate whether a gender difference exists in the association.

### Sample and registers

The study sample consisted of respondents to the North Denmark Region Health Survey 2010. The survey contained the “Short Form 12-item Survey” version 2 (SF12) as well as 22 items related to social capital, in addition to a large number of health-related questions. The sampling for participants used a municipality stratified random sampling strategy and in total 35,700 residents, above the age of 16, were sampled from 579,000 inhabitants in 11 municipalities. The survey was distributed in paper by mail in February 2010, and both paper and online responses were possible. Additional reminder letters were sent to nonresponders twice. The North Denmark Region Health Survey 2010 had 23,392 respondents (65.5%). Of these, eight persons died before the start of the follow-up period, resulting in a final sample size of 23,384 persons. Detailed information on response rates and the population is available elsewhere [26, 27]. All respondents were followed from March 31, 2010 until December 31, 2012 or death; this constitutes a follow-up period of 148 weeks.

The unique civil registration number assigned to each Danish resident allows individual linkage of survey data with information from different administrative registers [28].

In addition to the aforementioned survey data, the study used data from: The Income Statistics Register, which is a nationwide registry containing data on the taxable income of Danish residents [29], the Population Education Register, which contains information on the highest attained educational level of residents in Denmark [30], the Danish National Health Service Register, which contains data on all services billed by private health practitioners to the publicly funded Regions [31]. The ages and genders of the respondents were attained from the Danish Civil Registration Register [28].

### Variables

#### Frequent attendance

We operationalized health care utilization as any appointment that was marked as a consultation with a general practice in the Danish National Health Service Register. A consultation is marked when the appointment requires personal contact and includes both face-to-face contacts and email and telephone consultations. We dichotomized this variable into frequent attenders, who constituted the upper quartile of utilization (> 32 consultations over the 148 week period), and others. A number of different cutoff values are used in the literature in regards to operationalizing frequent attendance, the current approach was chosen on the basis of previous literature [10] and different cutoffs are tested to ensure the robustness of the operationalization.

### The social capital measures

Based on the approach used by Ejlskov et al. [18], four dimensions consisting of two cognitive dimensions (perceived norms of reciprocity, interpersonal trust) and two structural dimensions (social network and civic engagement) were used in the main analysis (Table 1). The North Denmark Region Health Survey 2010 contained several items related to the social life of the respondents, only a selection that were in line with the approach used by Ejlskov et al. [18] were analyzed in the current study. All items were centered and standardized to account for different response scales, this was done using the scale function of R, and the final scale score was defined as the average of the z-score of the constituting items. This resulted in a numeric score for each social capital scale, that weighs each constituting item equally, whether they have three, five or n response levels.

**Table 1:** Theoretical components of social capital and the constituting items from the Danish Health Profile 2010 survey

Dimension	Question	Categories
Interpersonal Trust	Indicate your agreement with the following statement: “Most people can be trusted.”	1: Completely disagree 2: Disagree 3: Agree 4: Strongly agree
	Indicate your agreement with the following statement: “You cannot be too careful when dealing with other people.”	1: Completely disagree 2: Disagree 3: Agree 4: Strongly agree
	“How much trust do you have in A: Your family / B: Your colleagues / C: People from your neighborhood / D: First time acquaintances / E: People of a different religion / F: People of a different nationality”^a^	For each group: 1: Complete trust 2: A fair amount of trust 3: Little trust 4: No trust
Perceived norms of reciprocity	“Do you have someone to talk to when you experience problems or are in need of support?”	1: Yes, often 2: Yes, mostly 3: Yes, occasionally 4: No
	“If you experience illness and need practical help (cooking, shopping, cleaning, dressing etc.), can you count on the help of others?”	1: Yes 2: Maybe 3: No
Social network	“How often do you interact with A: Friends / B: Acquaintances / C: Family you aren’t currently living with?”^a^	For each group: 1: Daily or almost daily 2: Once or twice per week 3: Once or twice per month 4: Less than once per month
	“Have you used the following local facilities/attended the following events A: Cinema, theatre or concert venues / B: Cafés, restaurants or nightclubs / C: Events, such as jogging, town festival, health days, and night events etc. within the last month?”^a^	For each facility: 1: Yes 2: No
Civic engagement	“Are you involved in associations/voluntary work or similar activities? A: Within the city you live in / B: Outside of the city you live in”^a^	For each activity: 1: Daily or almost daily 2: Once or twice per week 3: Once or twice per month 4: Less than once per month 5: Never
	“Have you used the following local facilities A: Church, congregational activities, mosques, synagogues / B: Clubs for the elderly / C: Youth clubs or similar / D: Town or village hall/ community center within the last month?”^a^	For each facility: 1: Yes 2: No

^a^ Letters indicate separate items. The original questionnaire is in Danish, the translations are by the main author

### Socio-demographic variables

The highest attained educational level for each individual in 2010 was subdivided into 3 groups based on International Standard Classification of Education (ISCED) codes [32]; “Lower than upper secondary education” (ISCED level 0–2), “Upper secondary education/vocational training” (ISCED level 3) and “Bachelor degree and higher” (ISCED level 4+).

Income was based on personal income in 2009 and divided into groups based on quartiles. For income group 1, the income was below DKK 184,623 (~ USD 31,600), for group 2, it ranged between DKK 184,624 and DKK 318,504 (USD 31,600–54,600), group 3 included respondents with an income between DKK 318,505 and DKK 444,411 (USD 54,600$-76,100), and group 4 included those with an income above DKK 444,411 (USD 76,100). All dollar values were calculated at 5.84 DKK/USD (July 2010 exchange rate).

### Self-reported health

The scoring of the SF12 follows the norm-based approach outlined in the SF-12v2 User’s Manual, using the original sample norms from 1998 US [33]. The 12 items included in the SF12 is combined into summary scores according the guidelines of the User’s Manual. Each respondent was thus assigned both a physical and mental health summary score. The SF12 has previously been validated in a Danish context, showing a Cronbach’s α score of 0.90 for the physical health component summary score and 0.85 for the mental health component summary score [34]. In the final model of analysis, we adjust for both the mental and physical health summary scores.

### Statistical analysis

Controlling for interaction in the initial models confirmed the relationship between gender and social capital; therefore the primary analysis is stratified by gender.

Descriptive statistics of the sample (Table 2) were conducted for each gender.

**Table 2:** Descriptive statistics of respondents to the North Denmark Region Health Survey 2010. For categorical variables, n and column percent are presented. For continous variables, mean and SD are presented. Results are stratified by gender

Variable	Level	Female (*n* = 12,097)	Male (*n* = 11,287)	Total (*n* = 23,384)	P-val
Age	Mean (sd)	50.8 (18.2)	50.8 (18.0)	50.8 (18.1)	0.769
Educational Level	Primary education	4446 (37.6)	3765 (34.1)	8211 (35.9)	
	Upper secondary / vocational training	4440 (37.6)	5184 (47.0)	9624 (42.1)	
	Bachelor’s degree and higher education	2935 (24.8)	2084 (18.9)	5019 (22.0)	< 0.001
	Missing	276	254	530	
Income quartile	Lowest Quartile	3149 (26.0)	2698 (23.9)	5847 (25.0)	
	Second Quartile	3046 (25.2)	2798 (24.8)	5844 (25.0)	
	Third Quartile	2953 (24.4)	2893 (25.6)	5846 (25.0)	
	Highest Quartile	2949 (24.4)	2898 (25.7)	5847 (25.0)	< 0.001
SF 12 physical component summary score	Mean (sd)	48.8 (10.9)	49.6 (9.5)	49.2 (10.2)	< 0.001
	Missing	1933	1362	3295	
SF12 mental component summary score	Mean (sd)	55.2 (9.8)	57.2 (8.8)	56.2 (9.4)	< 0.001
	Missing	1933	1362	3295	
Number of general practice consultations	median (interquartile range)	21 (25)	13 (21)	17 (24)	

The association between the four dimensions of social capital and frequent attendance within 148 weeks of follow-up was explored through logistic regression. The unadjusted odds ratio (model 1) was estimated for each of the four social capital dimensions in individual models. Model 2 included age, education and income as potential confounders. Model 3 included the same variables as model 2, with the addition of the physical and the mental health component scores of the SF12.

To minimize a potential bias due to missing values (Additional file 1), multivariate imputation by chained equation (MICE) [35] was used. MICE is generally the preferred method of imputation when missing values occur in several different types (i.e. categorical, continuous etc.) of variables in the data set [36]. The method used for prediction was Random Forest, a machine learning technique well suited to handle nonlinearities and high dimensional data [37, 38]. Nonlinearities were suspected due to the complex relationship between gender and social capital. The imputation model featured all variables included in analysis models, as well as several additional variables from the original dataset, to satisfy the “missing at random” assumption [36]. The R code used for the imputation and a list of variables used are available in Additional file 1.

All results are reported as odds ratio with an estimated 95% confidence interval. The primary analysis was carried out on the imputed data, and the results reported are pooled according to Rubin’s rules [39].

In addition to the analysis outlined above, sensitivity models were run to investigate the robustness of the results. Row-wise deletion of missing data (complete case analysis) was tested as an alternative to imputation. As the stratified sampling procedure might have influenced the results, the survey package for R was used to calculate additional complete case estimates [40]. The dichotomization of frequent attendance was tested using division at each decile as the potential cut-off value; each cut off was tested in the unadjusted models.

Initial data management was conducted using SAS software, version 9.4 (SAS Institute Inc., Cary, North Carolina, USA). Data analysis was performed using the R statistical software package, version 3.3.2 [41]. The imputation was implemented through the MICE package, version 2.25 [35].

## Results

### Descriptive statistics

Descriptive statistics of the sample population are presented in Table 2. Both genders had an average age of 50.8 years. Women had a higher prevalence of primary education (37.6%) and university degrees (24.8%) than men, while men had more commonly attained upper secondary/vocational education (47.0%). Men had a higher probability of belonging to the two higher quartiles of income, corresponding to a higher average income. Men scored higher than women in both SF12 health measures, which means that male respondents had better self-reported health than their female counterparts. Women had an average of 27.3 consultations with a general practice during the 148-week period, in contrast to the 19.6 consultations for men.

### Association between social capital and frequent attendance

**Fig. 1:**
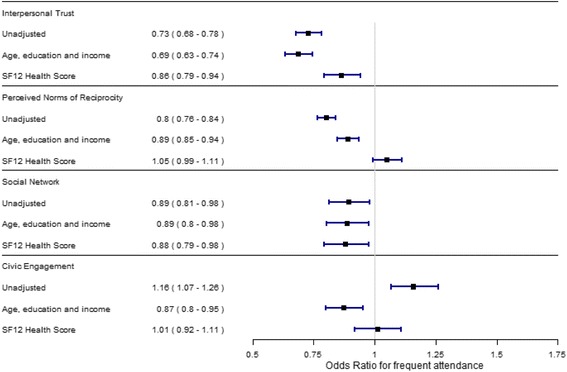
The results of the logistic regression analysis on the association between social capital dimensions and odds of frequent attendance for women

In the initial unadjusted models, higher levels of interpersonal trust, perceived norms of reciprocity and social network were statistically significant predictors of lower odds of frequent attendance for women (Fig. 1). Civic engagement showed a smaller, but statistically significant, positive prediction of frequent attendance among women.

**Fig. 2:**
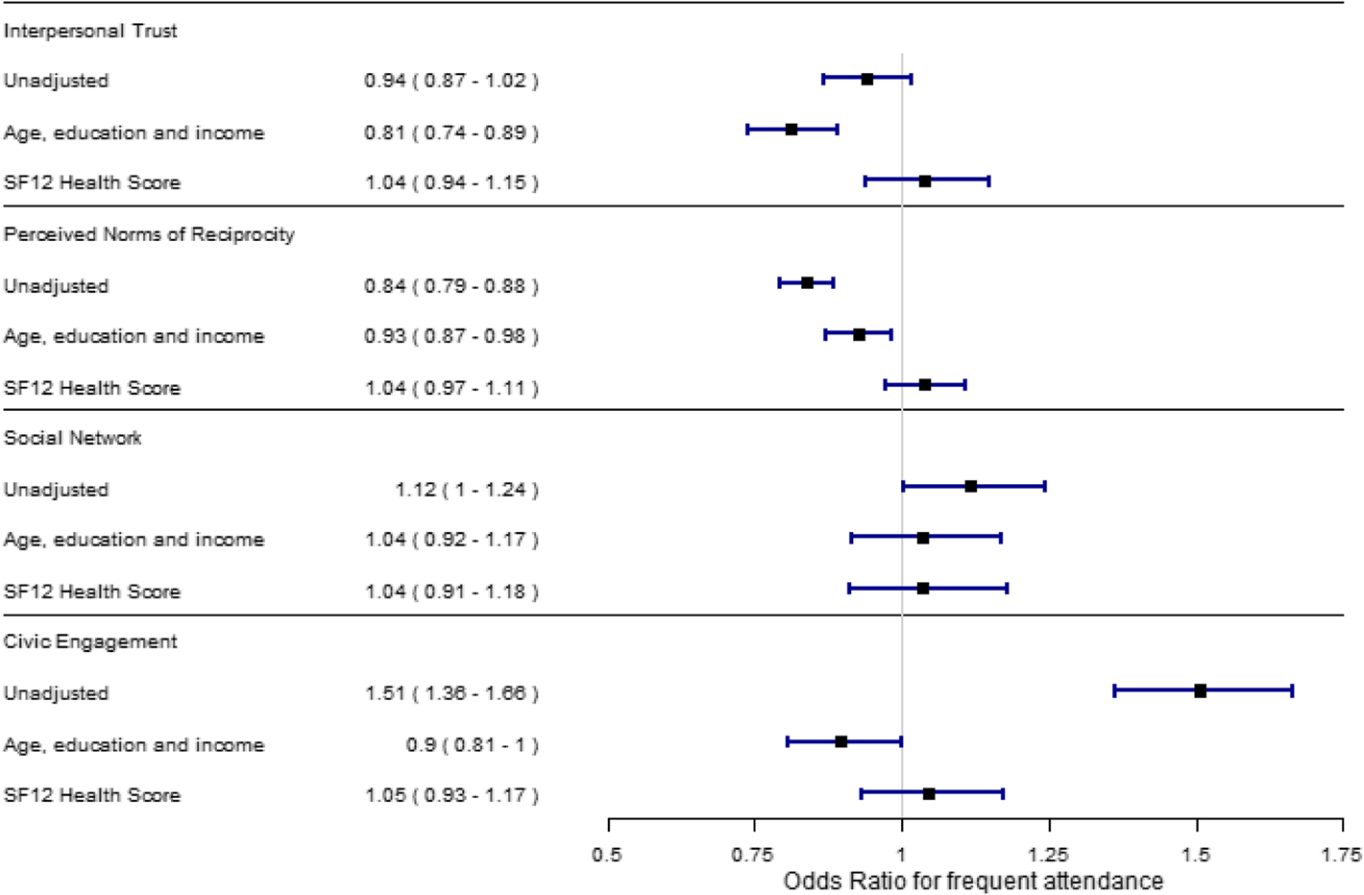
The results of logistic regression analysis on the association between social capital dimensions and odds of frequent attendance for men

For men (Fig. 2), perceived norms of reciprocity showed a negative association with frequent attendance, while social network and civic engagement showed a statistically significant positive correlation in the unadjusted model.

In model 2, which is adjusted for socioeconomic differences, all four dimensions were statistically significantly associated with lower odds of frequent attendance among women. Each standard deviation increase in interpersonal trust among women was associated with a 30% decrease in odds of belonging to the frequent attendance group. Similarly, each standard deviation increase in perceived norms of reciprocity, social network or civic engagement was associated with a decrease in odds of approximately 10%.

For men, interpersonal trust was associated with a 7% decrease in odds of belonging to the frequent attendance group per standard deviation increase; for perceived norms of reciprocity and civic engagement, the odds were decreased by 11% and 13%, respectively, per standard deviation increase in the social capital dimension when adjusting for socioeconomic factors. Social network was not statistically significantly associated with frequent attenders in the adjusted model.

Models were further augmented with the mental and physical scores of SF12, to adjust for self-reported health. For women (Fig. 1, model 3), a standard deviation increase in interpersonal trust was associated with a 14% decrease in odds of belonging to the frequent attendance group. Social network was associated with an estimated 12% decrease in odds. Perceived norms of reciprocity and civic engagement were not associated with the odds of being a frequent attender in the fully adjusted model. Among men, none of the dimensions were statistically significantly associated with frequent attenders when taking into account differences in demography and self-reported health.

### Sensitivity analysis

A wide range of cut-off values for frequent attendance were tested. The analysis turned out to be mostly insensitive to different cut-off points; however, the CI widened with more extreme cut offs (plots are available in Additional file 2). Moreover, the full model was tested on non-imputed data. Men in the non-imputed sample had a statistically significant positive association between interpersonal trust and frequent attendance (odds ratio: 1.19, 95% CI: 1.01–1.39). The association between the perceived norms of reciprocity and frequent attendance among women became statistically significant (odds ratio: 1.07, 95% CI: 1.00–1.14). Other results concurred with those of the main analysis and are available upon request. Additional analysis was performed on the nonimputed data, to investigate the effects of the stratified sampling procedure. Correcting for the stratified sampling did not change the estimates.

## Discussion

### Findings

This paper has examined the relationship between four dimensions of social capital representing structural and cognitive aspects and frequent attendance in general practice in a Danish health care setting. In our final models we found little evidence of a strong relationship between social capital and frequent attendance in primary care. Two of our social capital measures remained significant for women, but not for men, lending credence to the hypothesis that social capital has gender-specific effects.

### Gender-specific effects

With the exception of social network, all dimensions of social capital showed a statistically significant negative association with the odds of frequent attendance in men when the models were adjusted for sociodemographic characteristics, but not for self-reported health. In women, a negative association between social capital and frequent attendance was seen, even when accounting for self-reported health. This suggests that people with low social capital are more likely to be frequent attenders, which is in line with literature establishing the positive health effects of social capital [1]. The attenuating effect of adjusting for self-reported health for men suggests not only a relationship between health and frequent attendance, but also a correlation between self-reported health and social capital. This concurs with the findings of Subramanian et al. that increased social trust was correlated with better self-reported health [42]. Our findings show that men’s risk of frequent attendance is independent of their social capital, when accounting for self-reported health. This implies that men’s social capital includes resources different from those available at the general practice, which is in line with findings that men’s networks feature a larger proportion of non-kin contacts, typically signifying weak ties, which do not purvey the same emotional resources as close-knit family relations [2, 43].

### Interpretation of findings

Our results indicate that for women, increased social capital reduces the risk of being a frequent attender, independently of actual health status. Frequent attendance in general practice is often preceded by a need for health care services or health care advice; resources inherent in social capital might substitute the resources available from the practice, thus lowering attendance. These findings contrast those of Derose et al., who found that a majority of studies indicated a positive correlation between health care utilization which was dependent on need and social capital [6]. This contrast is, in part at least, explainable by the difference in utilization measures between the current study and the studies identified by Derose et al. We investigated a specific group of high-utilization patients, while the studies identified in the review investigated several other measures, including mental health services and self-reported health care access, across a broad range of populations [6]. It is possible that a different pattern of utilization and association with the social capital measures might be prevalent in the context of other forms of health care. This is corroborated by Danish health inequality studies, finding that patterns of the use of the health care system are consistent across socioeconomic strata in the general practice setting, but show inequality when factoring in specialist care; this applies in particular to care which requires financial copayment, which in the Danish setting includes dental care and prescription medication [44]. At the same time, the results might be skewed as a result of self-reported health being an insufficient proxy to capture all health information. People will experience a need for consultation based on both previously diagnosed existing disease and new disease and when they are feeling ill, and it is not easy to disentangle these “objective” and “subjective” causes. As the probability of obtaining an objective diagnosis will depend on any previous meeting with the general practice, we chose to use only the subjective part as our measure of health. Capturing all aspects would require detailed information on health and feelings of wellbeing that are not obtainable in a register-based approach.

The relatively small estimates of association between odds of being a frequent attender and social capital found in the current study suggest that the impact of social capital on high health care utilization might be modest in the Danish health care setting. This is in line with earlier findings, which indicated that psychosocial factors had very little impact on utilization patterns in Denmark [45]. This association might be different in other health care systems. With barriers of access to the general practice, such as finance, individuals might become more reliant on alternative sources of health care and advice, such as social capital. This would result in an unequal reduction in odds of frequent attendance between high and low social capital individuals and contribute to a larger degree of association.

Our findings support previous evidence that the influence of social capital on health behavior might differ between genders [21]. This might reflect a difference in the nature of social capital accessible to men as compared to that of women. Men generally have larger networks featuring fewer family ties than women [43]. Family ties might contribute differently to health-related behavior than more distant relations, corresponding to the theoretical divide of bonding and bridging social capital. It is possible that family relations provide an outlet for mitigating excessive health care use, due to the family’s role as both emotional and informational support provider. The difference in association between social capital and health care utilization between genders, might also be partly explained by their general difference in health behavior. Men often cite embarrassment and need for independence and control as barriers to health-seeking [23], and these barriers could potentially prevent men from realizing health related resources in their network. The nature of the survey questions makes it difficult to distinguish completely between bonding and bridging resources; however, both of the statistically significant dimensions predominantly measure the bonding aspects of social capital. Our results suggest that social capital interventions aimed at reducing the prevalence of frequent attendance should focus on strengthening this aspect rather than developing bridging resources.

### Limitations

We did not control for the impact of spouses in the current study; previous literature has indicated that men in a relationship are influenced by their partner in the context of health behavior [8, 46]. The partner’s role in modulating health behavior for men could explain the lack of an association between social capital and high health care utilization for men in the final model. This aspect could be explored with a more detailed dataset regarding partnership status and interactions. However, several of the items included in the social capital scales would be affected by partnership status, and we expect to have captured most of this information with the items used.

The analysis was conducted using previously gathered survey data, hence two important considerations are the validity of the items and the response rates. The items used in the survey were explicitly aimed at investigating social capital; however, they did not correspond to any accepted scale, but rather incorporate questions from several sources.

It is possible that the dichotomizing of health care utilization into frequent attenders and others influences the measured association. Dichotomization can lead to loss of information and in particular loss of statistical power [47, 48]. Thus, we may have found wider confidence intervals than if we had used a continuous measure of utilization; however, our estimates of association are close to one, and we would not expect our conclusion to change with a larger sample. Moreover, we examined different cut-off points, and this did not change the estimates.

Importantly, we did not investigate the direct causal pathways for social capital to interact with health care utilization. While others have examined this connection, little is known for certain [6]. A more comprehensive understanding of the mechanisms involved, might have helped in selecting a more specific measure of social capital, which could lead to a more firmly established association, or lack thereof, with health care utilization.

### Strengths

We have made an attempt to adhere to robust methodological choices and a consistent theoretical framework. Thus, we opted for a theoretical approach in the construction of the scales in order to provide results which were more interpretable to policy makers. Another benefit of this approach is that the dimensions are easily comparable to similar concepts in other studies. Another approach could be data-driven, incorporating statistical methods of dimension reduction such as PCA and factor analysis.

The large sample size of the study (*n* = 23,384) and the nature of Danish administrative registers allowed us to account for several potential confounders in the statistical models. In addition, the universal health care setting in Denmark facilitated the possibility to observe health care utilization, presumably independently of individual financial circumstances. While the original dataset featured a high number of missing values at item level, the use of imputation allowed the analysis to use the information from partial responses. While this presumes that the missing data is missing at random, this presumption is more lax than that of complete case analysis, which requires missing completely at random data for unbiased results [36, 49]. Thus, the methodological approach is based on recommendations in statistical literature, and we have sought to minimize any potential bias, to ensure the validity of our results.

This study was conducted explicitly at the individual level, whereas previous literature has featured multi-level or area-level analyses, which might influence the findings. While sound arguments exist for both measures, empirical evidence suggests that area-level effects are mediated through individual-level characteristics [50]. If the effects of social capital originate at the area level, we would expect them to be inherent in our individual-level measures.

### Implications and future research

We found some evidence of the association between social capital and health care utilization for women, which might imply a potential point of intervention for addressing problems with frequent attendance in women. However, the need for establishing the pathways that facilitate this relationship, might not be solvable through register- and survey- based cohort studies such as this one, and might instead benefit from a qualitative approach, as the pathways might differ on an individual level. This might also lead to new insight into the qualitive differences in men and women’s social capital. We found no evidence of an association between social capital and health care utilization for men and for public health researchers and interventionists, this difference between genders is important to consider when planning future interventions in social capital, as a differentiated approach might increase the effectiveness of the intervention.

## Conclusion

This study suggests that for women, some aspects of social capital are associated with frequent attendance in general practice. This association was not seen for men. This indicates a multifaceted and heterogeneous relationship between social capital and frequent attendance among genders.

## Additional files

Additional file 1: Missing Data and Imputation Procedure. (DOCX 21 kb)

Additional file 2: Sensitivity Analysis. (DOCX 88 kb)

## Abbreviations

DKK: Danish Kroner
ISCED: International Standard Classification of Education
MICE: Multiple Imputation by Chained Equations
SF12: Short Form 12 Item Survey version 2
USD: United States Dollars
