# Supplementary material for: Social capital and frequent attenders in general practice: a register-based cohort study
Source: BMC Public Health. 2018 Mar 2;18:310. doi: 10.1186/s12889-018-5230-2 (PMC5834840; doi:10.1186/s12889-018-5230-2)
Supplement: Supplementary file 1 — Missing Data and Imputation Procedure. (DOCX 21 kb) [file 12889_2018_5230_MOESM1_ESM.docx]

# Additional file 1 – Missing Data and Imputation Procedure

Table of item level missing values.

A1.1 Imputation Procedure

A1.1.1 Variables

List of Variables included in the imputation procedure

From administrative registers:

Age, Gender, Number of consultations, Income Category, Education level, Time followed, Death during follow-up.

Questions from The North Denmark Health Profile 2010 (All items):

Q1, Q2, Q3, Q4, Q5, Q6, Q7, Q16, Q17, Q50, Q51, Q52, Q54, Q56, Q57, Q58, Q60, Q61, Q62, Q63, Q64, Q66(item a omitted), Q71, Q72

A1.1.2 Code

Imputation code (R statistics) with parameter values:

library(mice)

Imputed.data <- mice(Original.data,

meth=”rf”, #Method is set to Random Forest

ntree=10, #Number of trees

m=20, #Number of iterations

seed=3636) #Fixed random seed
