# Supplementary material for: Social capital and frequent attenders in general practice: a register-based cohort study
Source: BMC Public Health. 2018 Mar 2;18:310. doi: 10.1186/s12889-018-5230-2 (PMC5834840; doi:10.1186/s12889-018-5230-2)
Supplement: Supplementary file 2 — Sensitivity Analysis. (DOCX 88 kb) [file 12889_2018_5230_MOESM2_ESM.docx]

# Additional file 2 – Sensitivity Analysis

A2.1 Dichotomization

Plots of different cut-offs for frequent attenders.

A2.1.1 Men

A2.1.2 Women
